# Neural regulation in tooth regeneration of *Ambystoma mexicanum*

**DOI:** 10.1038/s41598-020-66142-2

**Published:** 2020-06-09

**Authors:** Aki Makanae, Yuki Tajika, Koki Nishimura, Nanami Saito, Jun-ichi Tanaka, Akira Satoh

**Affiliations:** 1 Okayama University Research Core for Interdisciplinary Sciences (RCIS), 3-1-1, Tsushimanaka, Kitaku, Okayama, 700-8530 Japan; 2 Gunma University, Department of Anatomy, Graduate School of Medicine 3-39-22 Showa-machi, Maebashi, Gunma, 371-8511 Japan

**Keywords:** Cell proliferation, Morphogenesis, Differentiation

## Abstract

The presence of nerves is an important factor in successful organ regeneration in amphibians. The Mexican salamander, Ambystoma mexicanum, is able to regenerate limbs, tail, and gills when nerves are present. However, the nerve-dependency of tooth regeneration has not been evaluated. Here, we reevaluated tooth regeneration processes in axolotls using a three-dimensional reconstitution method called CoMBI and found that tooth regeneration is nerve-dependent although the dentary bone is independent of nerve presence. The induction and invagination of the dental lamina were delayed by denervation. Exogenous Fgf2, Fgf8, and Bmp7 expression could induce tooth placodes even in the denervated mandible. Our results suggest that the role of nerves is conserved and that Fgf+Bmp signals play key roles in axolotl organ-level regeneration. The presence of nerves is an important factor in successful organ regeneration in amphibians. The Mexican salamander, Ambystoma mexicanum, is able to regenerate limbs, tail, and gills when nerves are present. However, the nervedependency of tooth regeneration has not been evaluated. Here, we reevaluated tooth regeneration processes in axolotls using a three-dimensional reconstitution method called CoMBI and found that tooth regeneration is nerve-dependent although the dentary bone is independent of nerve presence. The induction and invagination of the dental lamina were delayed by denervation. Exogenous Fgf2, Fgf8, and Bmp7 expression could induce tooth placodes even in the denervated mandible. Our results suggest that the role of nerves is conserved and that Fgf+Bmp signals play key roles in axolotl organ-level regeneration.

## Introduction

The ability to regenerate lost tissues or organs in human patients has long been eagerly desired. Teeth are among the organs for which a novel treatment allowing perfect regeneration would be extremely useful. Due to the unique and multifarious roles of the teeth and associated structures, there has been a sustained effort to find optimal ways to replace missing dentition over many centuries.

Amphibian teeth have unique physiological features but maintain some structural and functional similarities to mammalian teeth^1,2^. One of the most notable features of amphibian teeth is polyphyodonty. Through polyphyodonty, amphibians replace their teeth continuously during life, whereas each mammalian tooth has either one or two generations (mono- or diphyodonty). Amphibian tooth development has been described in some classic studies^3–10^. As far as we know, Hertwig published the first study of amphibian tooth development^5^. Recently, the literature on tooth regeneration in amphibians was reviewed^1^. The dental lamina is induced from the oral epithelium. The dental lamina, which consists of two layers, invades into the oral mesenchyme. The distal region of the dental lamina interacts with mesenchymal cells and forms a cup that is composed of the outer dental epithelium and the inner dental epithelium. The inner dental epithelium differentiates into enamel organs. The mesenchymal cells facing the inner dental epithelium differentiate into a dental papilla. The dental papilla cells then further differentiate into odontoblasts. Dentine and predentine are deposited between the ameloblast layer, which is differentiated from the inner dental epithelium, and the odontoblasts. The ameloblasts deposit a thin layer of an enamel-like matrix called enameloid on the surface of the dentine, and thus a new tooth is formed. The teeth thus formed two distinct positions within the tooth alignment: they can be attached to the paired bones of the lower jaw (dentaries and coronoids, functional tooth), or they can remain detached from the jaw bones, in which case they are easily lost (non-functional teeth). Rows of these teeth are induced on the dentary and coronoid bones. In newts, the coronoids disappear during metamorphosis. In axolotls, however, the coronoids remain because of the neotenic life cycle. Axolotls undergo a tooth replacement cycle in which reserved tooth buds are lined up along the base of each functional tooth^1^. These reserved tooth buds are in earlier stages of tooth development compared to the functional teeth. This allows us to investigate the various stages of tooth development within a single animal. Thus, axolotl dental developmental processes and structures are similar to those in mammals, but their polyphyodonty makes it much easier to study their teeth.

A systematic and histological description of salamander tooth regeneration was made about half a century ago^11,12^. In that report, tooth regeneration was described as a part of jaw regeneration. After partial removal of the lower jaw, the emergence of new teeth can be observed. These newly forming teeth can be seen as soon as wound healing has finished. Indeed, tooth regeneration takes place long before jawbone regeneration does. Tooth buds are induced from the inner layer of the buccal epithelium, i.e., the dental lamina. Previous reports have strongly suggested that tooth bud regeneration depends on the residual lamina in the stump region^13–15^. While these experimental descriptions represented a thorough investigating for their time, tooth regeneration has not been revisited with current biological technologies.

In this study, we revisited amphibian tooth regeneration using current histological and molecular biological techniques. We obtained three-dimensional (3D) morphological data on the jaw of the axolotl, *Ambystoma mexicanum*, and clearly identified two types of teeth: teeth attached to the dentaries or coronoids, and isolated teeth. In addition, we analyzed tooth regeneration after injury. 3D morphological and immunofluorescence analysis revealed the morphology of the regenerating tooth and the distribution of the nerves serving the regenerating tooth. We found that denervation prevented tooth regeneration, and that overexpression of Fgf2, Fgf8, and Bmp7 could induce a dental placode in a denervated mandible. Our results reveal the molecular mechanisms underlying tooth regeneration in axolotls, which may be applicable to other vertebrates.

## Results

### Axolotl tooth structure and replacement

**Figure 1:**
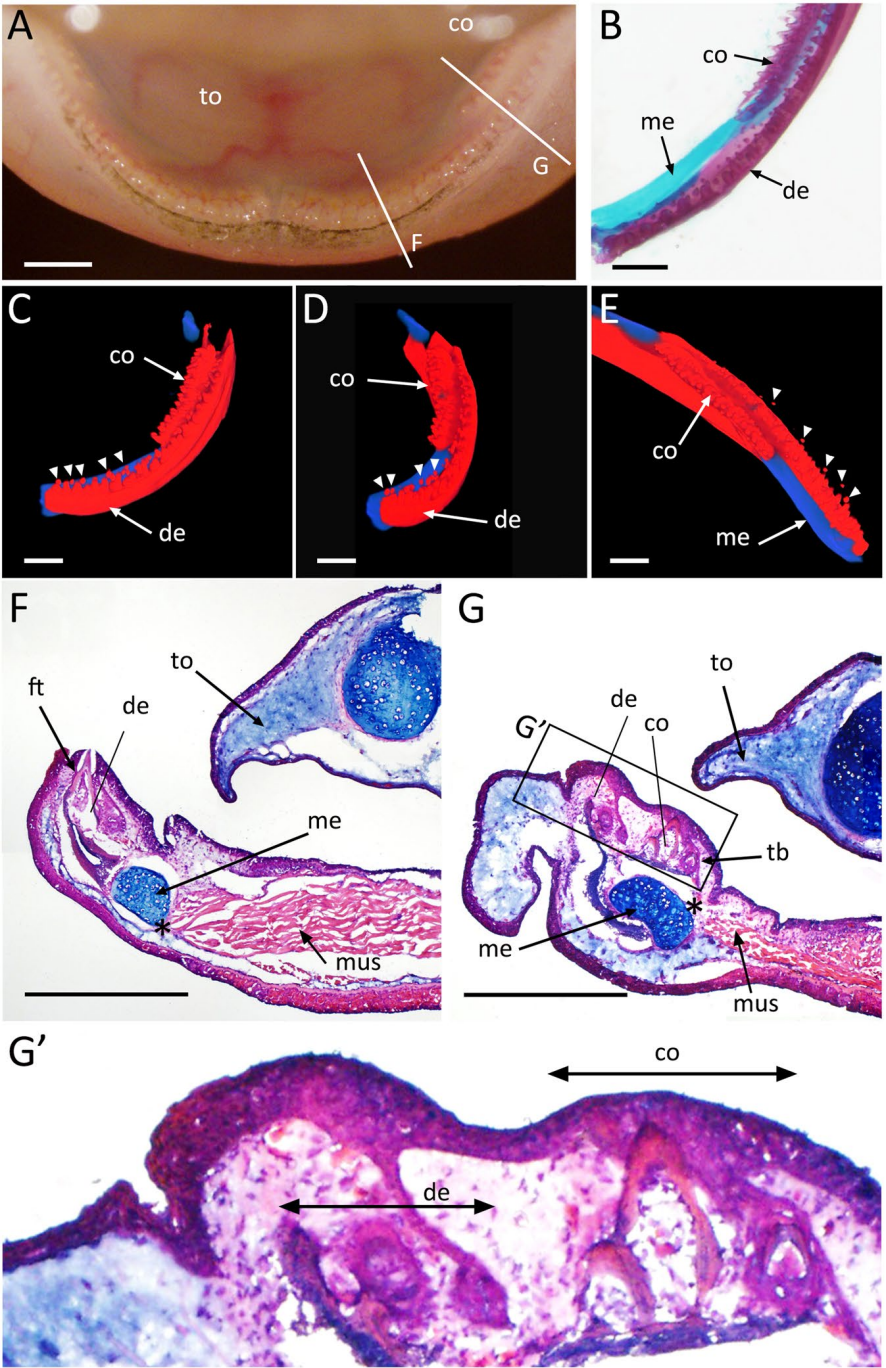
Axolotl mandibular structure. (**A**) Axolotl mandible. (**B**) Histological observation of the axolotl mandible. Alizarin red and Alcian blue staining show bones in red and cartilages in blue. The medial line is on the left and the lateral side is on the right. (**C–E**) CoMBI imaging. Bones and cartilage are shown in red and blue, respectively. The left mandible shown in B was sectioned and reconstructed into 3D images. Arrowheads indicate rootless teeth. Data are representative of n = 5 of axolotl mandibles. (**F, G**) Histological observation of the axolotl lower jaw. The approximate section lines are indicated in Fig. 1A. G’ is the higher magnification view of the boxed region in G. Asterisks indicate the connecting region between the Meckel’s cartilage and muscles. Hematoxylin/Eosin/Alcian blue staining was performed on the sections indicated in A. co = coronoid. de = dentary. ft = functional tooth. mus = Muscles. me = Meckel’s cartilage. tb = tooth bud. to = tongue. Scale bars = 1 mm. Data are representative of n = 8 of axolotl mandibles.

First, axolotl tooth histology and morphology were reexamined. The axolotl’s teeth are found on four independent bones, the two dentaries and the two coronoids (Fig. 1A,B). We further analyzed the mandibular skeleton using 3D imaging. Histologically stained samples were imaged using a CoMBI imaging system^16^. We could generate 3D structures of stained mandibular samples (Fig. 1C-E). Meckel’s cartilage is maintained even in adult axolotls (Fig. 1C–E). Teeth are lined up on the dentaries but the tooth line(s) on the coronoids is disturbed (Fig. 1C–E, Movie 1). The teeth that are lined up on the mandibular bones usually have small tooth buds on the basal region (Fig. 1C–D). It is particularly worth noting that some rootless teeth could be seen (Fig. 1A–E, arrowheads). Rootless teeth were always small, and no attachment could be confirmed at the bottom of any rootless tooth. For this reason, the rootless teeth were easily lost. Histological observation on sections revealed that the Meckel’s cartilage is connected with the muscles (Fig. 1F,G, asterisks). The teeth were attached to the dentaries and the tip of each tooth was exposed (Fig. 1F). At the coronoid level, the Meckel’s cartilage runs between the dentary and the coronoid (Fig. 1G). The dentary holds the attached tooth and the tooth bud on the inner side (Fig. 1G). On the coronoid, however, the tooth alignment is disturbed. The dentary and the coronoid are thin structures without bone marrow at this point (Fig. 1G). Replacement tooth buds on the coronoid were identifiable in our sections (Fig. 1G,G’).

**Figure 2:**
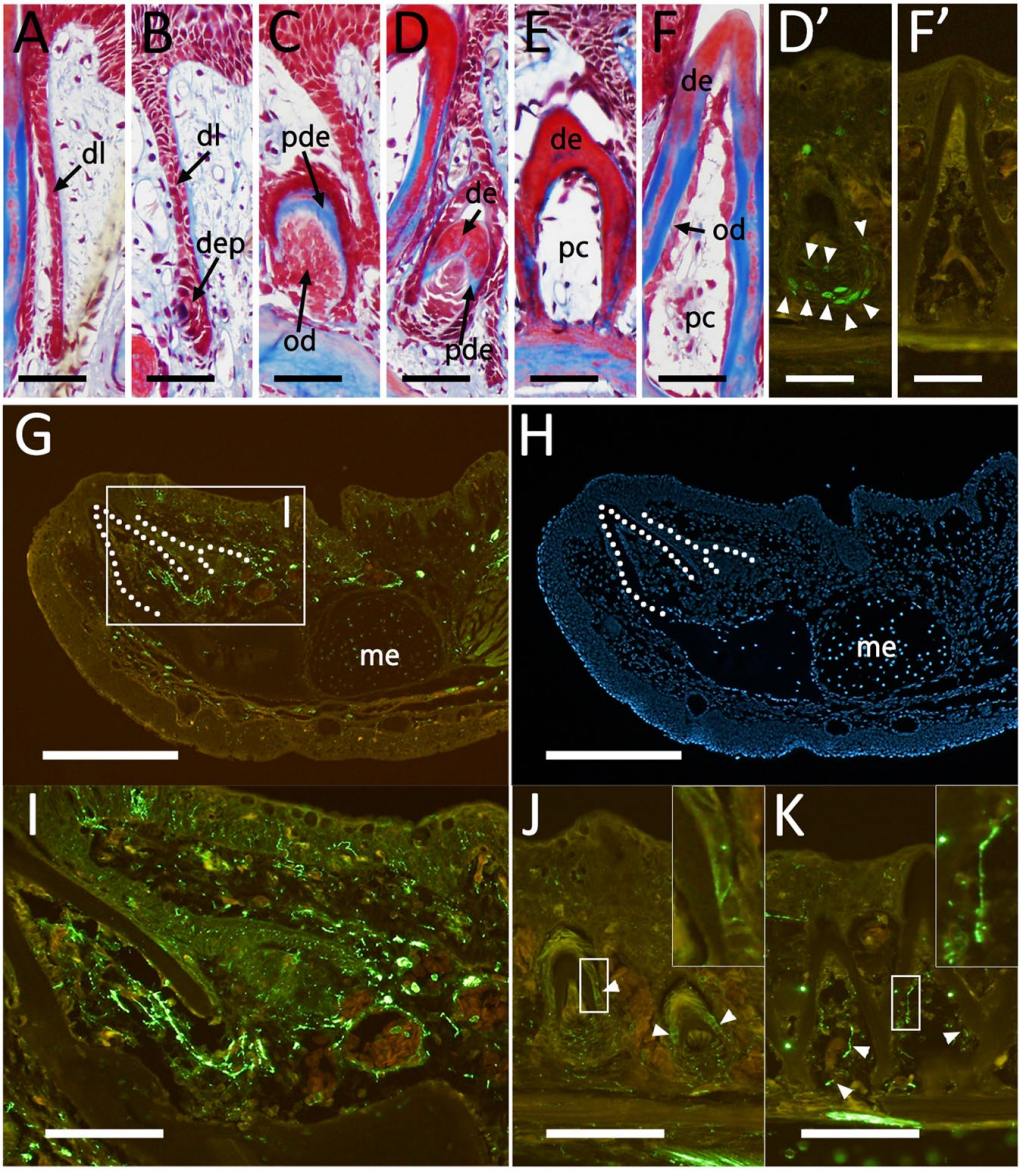
(**A–F**) Histological observations of the axolotl tooth. Masson’s trichrome staining visualizes collagen fibers in blue, cytoplasm in red and nuclei in dark purple. dl = dental lamina. dep = dental epithelium. od = odontoblasts. pde = predentine. de = dentine. pc = pulp cavity. (**D’ F’**) Cell proliferation was visualized using BrdU staining on the adjacent sections of D and F, respectively. Arrowheads in D’ indicate the BrdU-positive cells. (**G–K**) Nerve presence was visualized using anti-acetylated tubulin antibody. H shows Hoechst33342 signals on the same section depicted in G. Arrowheads in J and K indicate the Acetylated tubulin-positive cells in the developing tooth buds. I is the higher magnification view of the boxed region in G. The insert in J and K shows higher magnification views of the respective boxed regions. The dotted lines in G and H indicate a functional tooth and a tooth bud. dl = dental lamina. dep = dental papilla. pde = predentine. de = dentine. od = odontoblast. pc = pulp cavity. me = Meckel’s cartilage. Scale bars in A–F, and I–K are 200 μm. Scale bar in G and H is 500 μm. Data are representative of n = 4.

The polyphyodonty of axolotls allows us to see various stages of tooth development within a single sample. Tooth development is initiated by invagination of the inner layer of the dental epithelium. Two thin layers invaginate into the oral mesenchyme (Fig. 2A). At the end of these invaginated layers, a cup formation develops (Fig. 2B). The cells facing the mesenchymal cells differentiate into a dental papilla (Fig. 2C). The dental papilla cells generate odontoblasts, which deposit the predentine matrix (Fig. 2C). The predentine then mineralizes to become dentine (Fig. 2D). Cell proliferation can be observed in the basal portion of the odontoblast population by this stage (Fig. 2D’). Meanwhile, the tooth has elongated toward the surface of the oral epithelium (Fig. 2E). At this stage, the pulp cavity becomes apparent. The attachment to the mandibular bone is strengthened by connective tissues (Fig. 2E). A functional tooth is shown in Fig. 2F. No cell proliferation was detected inside the tooth (Fig. 2F’). Each functional tooth is well innervated. Axons, visualized by Acetylated α-tubulin antibody, are readily apparent in the section (Fig. 2G,H,K). The developing tooth buds were also targets of innervation (Fig. 2I,J,K), and nerves were present throughout this developmental process.

### Dentectomy and tooth regeneration

**Figure 3:**
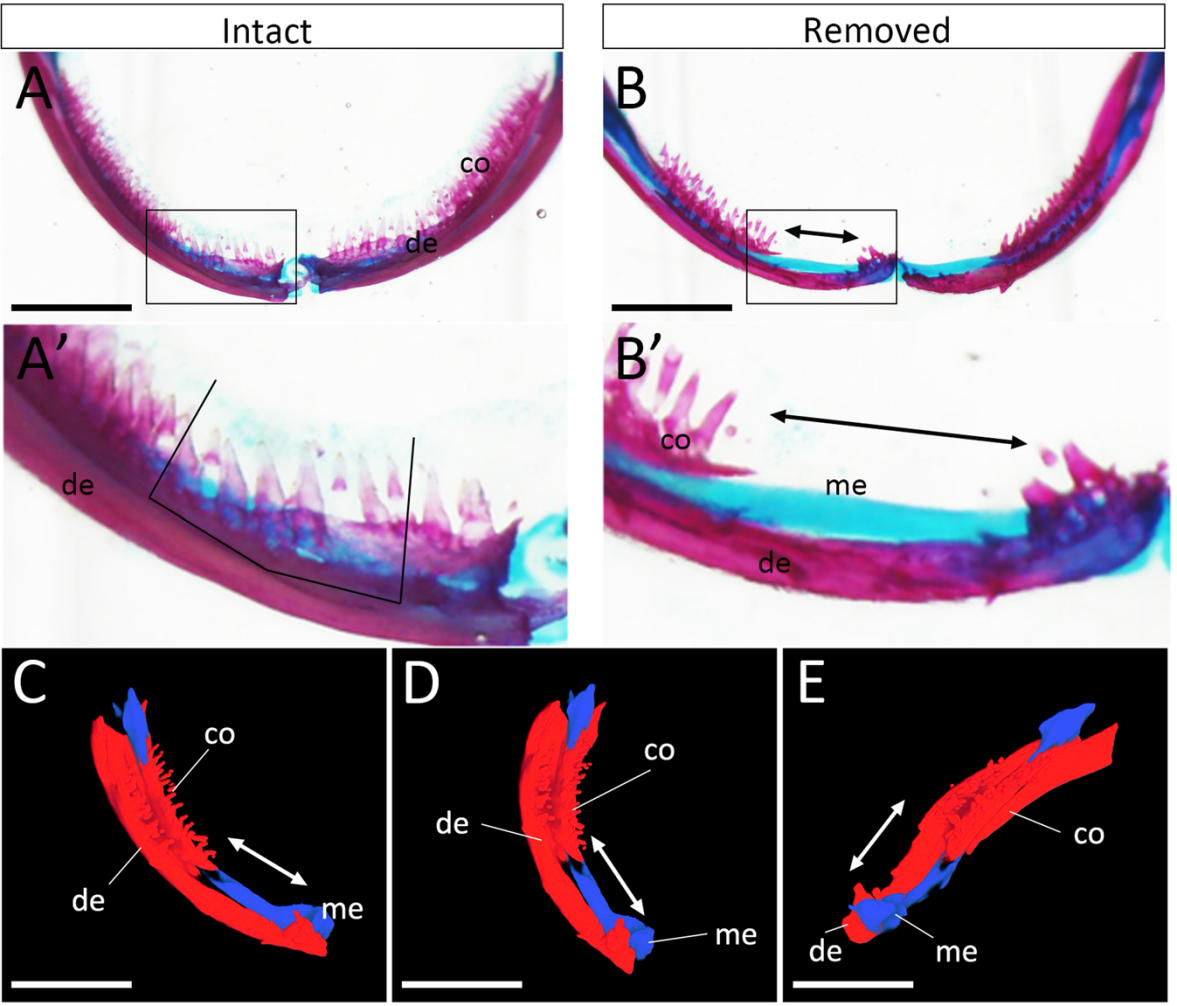
Dentectomy of the axolotl mandible. (**A,B**) Skeletal preparation stained with Alcian blue and Alizarin red. (**A, A’**) Axolotl mandible before dentectomy. A’ is the higher magnification of the boxed area in A. Lines indicate the approximate incision sites. (**B, B’**) Axolotl mandible just after dentectomy. B’ is the higher magnification of the boxed area in B. Arrows in B and B’ indicate the dentectomized area. co = coronoid. de = dentary. me = Meckel’s cartilage. Data are representative of 21 samples. (**C–E**) CoMBI imaging of the dentectomized mandible. Arrows indicate the dentectomized region. Scale bars = 500 μm. Data are representative of 3 experiments.

To investigate tooth regeneration in an axolotl mandible, dentectomy was performed by removing the dorsal half of the dentary (Fig. 3, Movie 2). The tooth on the dentary is lined up and easy to target, and there are many detouched teeth and tooth buds (Fig. 3A,A’; Fig. 1). To ensure perfect removal of the tooth organs, including the oral epithelium, the dorsal side of the oral tissues was removed (Fig. 3B,B’). The dentectomized mandible was reconstituted into a 3D image (Fig. 3C–E). The absence of the dorsal half of the mandible is apparent in these images in contrast to those shown in Fig. 1C–E.

**Figure 4:**
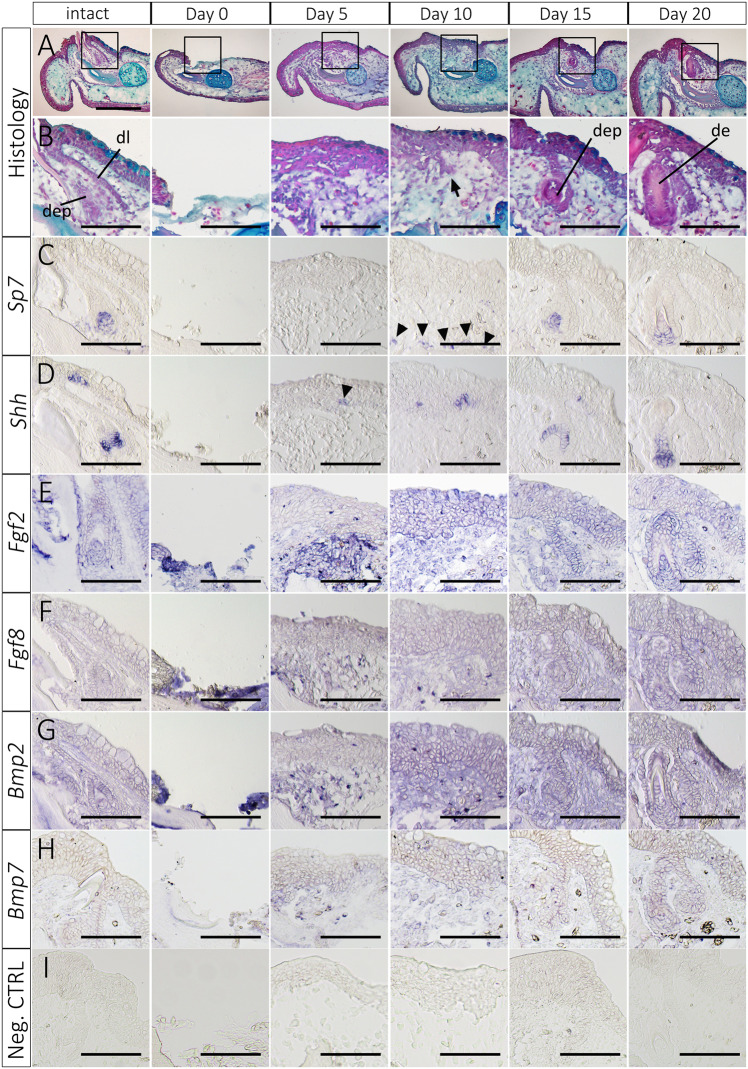
Gene expression patterns in the regenerating tooth. (**A,B**) Histological observations in the regenerating tooth. The images in rows B-H are higher magnification views of the boxed regions in row A. Arrow in B indicates the initiation of the invagination of the oral epithelium. dl = dental lamina. dep = dental epithelium. de = dentine. (**C**) *Sp7* expression. (**D**) *Shh* expression. (**E**) *Fgf2* expression. (**F**) *Fgf8* expression. (**G**) *Bmp2* expression. (**H**) *Bmp7* expression. (**I**) Negative control. RNA probes of *green fluorescent protein* (*gfp*) were used. Scale bar in A is 500, and scale bars in A–I are 200 μm. Data are representative of 5 independent samples.

To observe the process of tooth regeneration in the axolotl mandible, we sectioned regenerating axolotl mandibles, and investigated their histology and gene expression patterns (Fig. 4). Histological observation revealed that oral epithelium covered the wound surface within 5 days after dentectomy (Fig. 4A,B). No invagination of the covering oral epithelium was observed at this time. Indications of the invagination of the oral epithelium were first found around 10 days after dentectomy (Fig. 4B, arrow). This invaginated epithelium then formed a tooth as in normal tooth development. *Shh* and *Sp7* genes were investigated through *in situ* hybridization (Fig. 4C,D). *Sp7*, which has been reported as a late dental marker in vertebrates^17^, was found in the tooth buds in the intact oral tissues (Fig. 4C,the leftmost column). The expression pattern of *Sp7* was consistent with *Sp7*’s role in odontoblast differentiation (Fig. 4C). In tooth regeneration, *Sp7* expression is observed in relatively later phases of tooth regeneration (Fig. 4C). It was first observed on day 10 around the mandibular bone (Fig. 4C, arrowheads). Later, *Sp7* was found in the tooth bud mesenchyme where it contributes to odontoblast differentiation. Thus *Sp7* is a mesenchymal marker gene. The other gene we examined, *Shh*, has been reported as a broad and reliable dental marker in tooth development^18,19^. Consistently, *Shh* expression was found as early as day 5 in the pre-migratory dental epithelium (Fig. 4D, arrowhead). *Shh* expression can be used to visualize the presumptive dental organs before epithelial invagination. By day 15, *Shh* expression was observed in the cup epithelium (Fig. 4D). *Shh* expression was maintained in the later phases of tooth regeneration. The epithelial expression pattern of *Shh* makes it a useful marker gene for visualizing the whole process of tooth regeneration in axolotls.

Fgf and Bmp have been identified previously as nerve factor entities in limb and tail regeneration in axolotls^20,21^. Ectopic Fgf and Bmp application can substitute for the presence of nerve by fulfilling the role of nerves in denervated axolotl limbs^20^. An equivalent functionality of Fgf and Bmp has been reported in multiple organs and species^20,22,23^. Accordingly, we focused on the expressions of these genes in our examination of tooth regeneration in axolotls. *Fgf2*, *Fgf8*, *Bmp2* and *Bmp7* were investigated in the regenerating axolotl jaw (Fig. 4E–H). All genes were broadly expressed in the axolotl mandible. No obvious localizations of gene expression were observed with regard to *Fgf2*, *Fgf8*, *Bmp2* and *Bmp7* in tooth regeneration (Fig. 4E–H). All four of these genes were also expressed in invaginating epithelial cells. Signals of *Fgf2*, *Fgf8*, *Bmp2* and *Bmp7* expression in the regenerating mandible were relatively ubiquitous, especially in comparison with the negative control (Fig. 4I). This gene expression pattern suggests the involvement of Fgf and Bmp signaling in tooth regeneration.

### Nerve dependency of axolotl tooth regeneration

**Figure 5:**
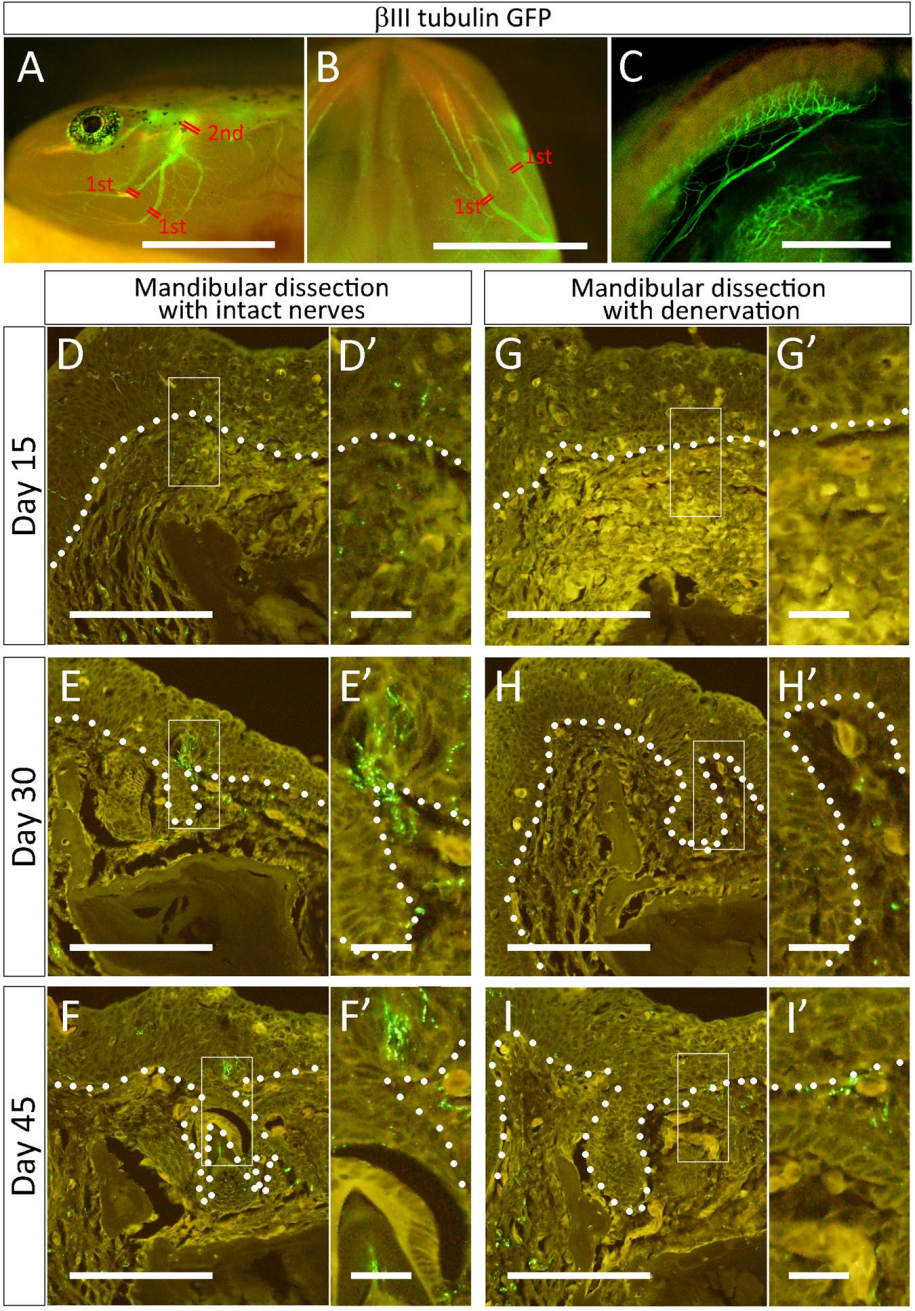
Nerve presence in the axolotl mandible and a regenerating tooth. βIII tubulin-GFP transgenic axolotl tissues show the presence of axons. (**A–C**) GFP signals in normal (control) axolotl mandible. (**A**) Lateral view. (**B**) Ventral view. (**C**) Dorsal view of the mandible. Subsequently, the lower jaw was dissected out and pictures were taken from the dorsal side. (**D–F**) Nerve presence in the regenerating tooth. GFP positive nerve fibers were visualized by anti-GFP antibody on the sections. D’–F’ are higher magnification views of the boxed regions in D–F. (**G–I**) Nerve presence in the regenerating tooth in the denervated mandible. G’–I’ are higher magnification views of the boxed regions in G–I. Scale bars in A, B, and C are 5, 5, 1. Scale bars in D–I and D’–I’ are 0.3 and 0.05 mm, respectively. The dotted lines indicate the border of the oral epithelium. Data are representative of 4 independent experiments.

To examine the structures of nerves in detail, we used βIII-tubulin GFP transgenic axolotls, which are convenient for monitoring axon presence in tissues^24^. Axons projecting to the mandible could be observed in the smaller specimens (Fig. 5). Axons projecting from the trigeminal ganglia toward the mandible were also visible (Fig. 5A–C). The nerves running into the ventral root exhibit a complex nerve projection pattern. In the proximal region of the dentary, the nerves branch apart into two major routes. In a βIII-tubulin GFP transgenic axolotl, GFP-positive fibers could be confirmed in the regenerating axolotl mandible on day 15 (Fig. 5D,D’). A few GFP-positive cells were identifiable in both the mesenchyme and the oral epithelium. The GFP signal was increased on day 30 (Fig. 5E,E’), and GFP signals were still found in the regenerated tooth on day 45 (Fig. 4F,F’). Notably, GFP positive axons could be seen in the epithelium adjacent to the forming tooth bud (Fig. 5E,E’,F,F’). This implies a positive relationship between tooth bud initiation and nerves. We next investigated the roles of nerves in tooth regeneration through denervation experiments. Our denervation procedure targeted the two major nerve routes in the mandibular region (Fig. 5A–C). In the first denervation, the proximal region of each branch was dissected (Fig. 5A,B). Dentectomy was performed on the same day as the first denervation. Knowing that newly regenerating axons emerge from the dissected ends of nerves and that these newly forming axons are invisible because of their thinness, we also performed a second denervation (on day 10) on the more basal region (Fig. 5A). In the denervated mandible, GFP signals were almost absent initially (Fig. 5G,G’). By day 30, however, a few GFP-positive fibers could be seen (Fig. 5H,H’). On day 45, the axon presence remained much lower in denervated mandibles than in control mandibles (Fig. 5F,F’,I,I’). These results indicate that our denervation procedure results in an aneurogenic state in the early phase but that innervation is somehow recovered in the later phases.

**Figure 6:**
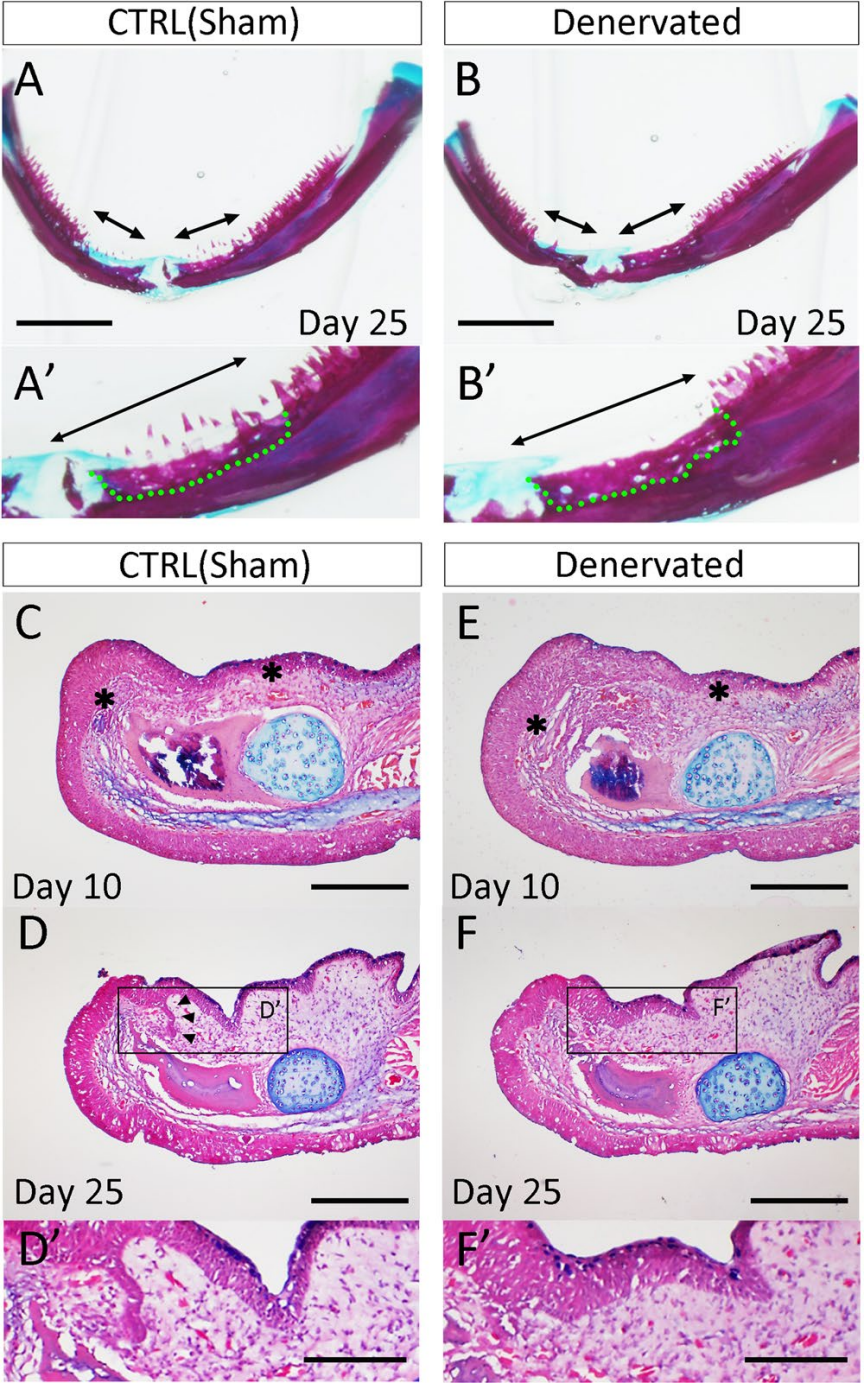
Tooth regeneration in innervated and denervated mandibles. (**A**) Alcian blue and alizarin red staining. Regenerating teeth could be observed in the sham-operated mandible. A’ is a higher magnification view of A. (**B**) Alcian blue and alizarin red staining. Regenerating teeth could not be observed in the denervated mandible. B’ is a higher magnification view of B. Arrows in A and B indicate the dissected regions and the green dotted lines indicate the regenerated dentary bone. Data are representative of 20 samples. Scale bars in A and B are 2 mm. (**C–F**) Histological observations of the regenerating axolotl mandible. Scale bars in C–F are 500 μm. (**C,D**) Sham-operated mandible. (**E, F**) Denervated mandible. The asterisks in C and E indicate the border of the intact tissues as determined by discontinuity of the oral collagen layers. This discontinuity disappeared on day 25 after the surgery. Arrowheads in D indicate the invaginating dental lamina. D’ and F’ are the higher magnification views of the boxed region in D and F, respectively. Scale bars in D’ and F’ are 200μm. Data are representative of 4 independent experiments.

Regeneration was assessed in innervated and denervated mandibles. On day 25, regeneration was apparent in the control (innervated) mandible and absent in the denervated mandible (Fig. 6). Of note, the dentary bones were well restored in both samples (Fig. 6A’,B’), though there were holes in the regenerated dentaries, suggesting that bone regeneration was still ongoing (Fig. 6A’,B’). The innervated dentary had regenerated teeth by day 25, though these regenerated teeth were immature compared to those in the intact region (Fig. 6A). In the denervated jaw, in contrast, the dentary was restored but the teeth were not regenerated (Fig. 6B). To investigate further, we made sections of the dentectomy samples and observed their histology (Fig. 6C–F). On day 10, no apparent differences were observed between the innervated and denervated samples (Fig. 6C,E). In control (innervated) samples, the invagination of the dental lamina became confirmable on day 25 (Fig. 6D,D’, arrowheads). In the denervated samples, in contrast, no sign of an invagination of the oral epithelium was observed (Fig. 6F,F’). The extension of the dentary, however, was noticeably advanced on day 25 compared to day 10. This finding indicates that nerve presence is important for tooth regeneration in axolotls.

**Figure 7:**
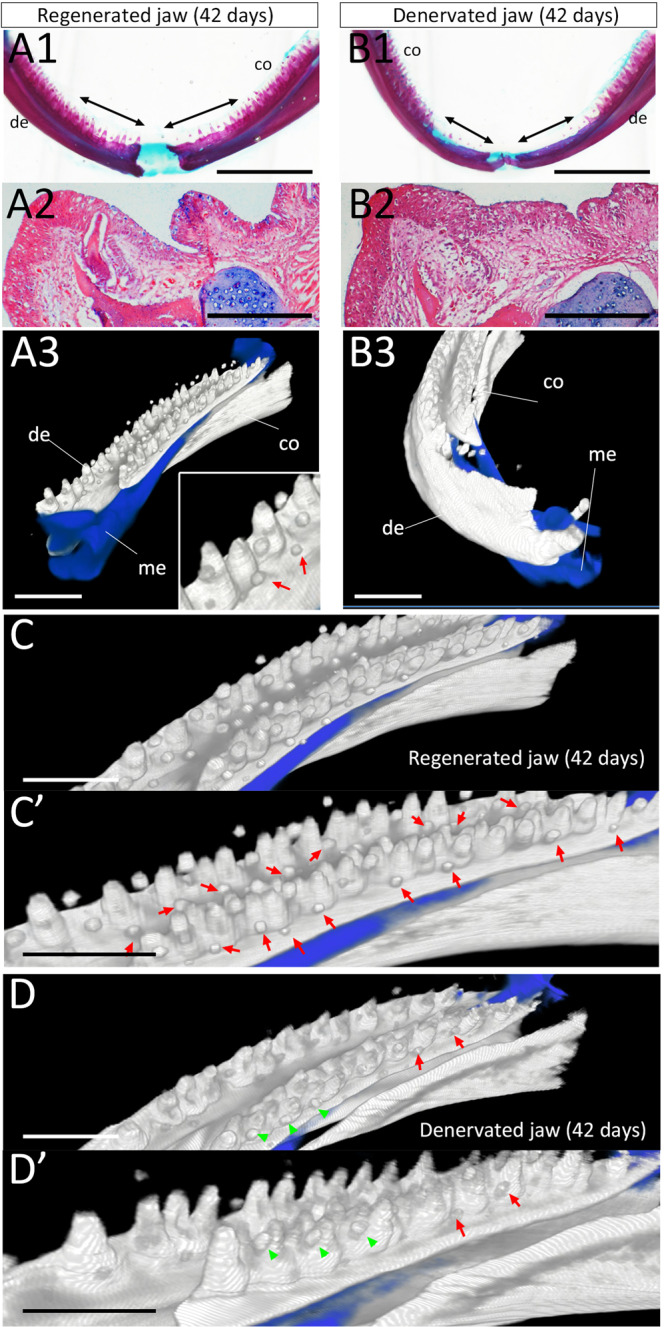
CoMBI imaging of tooth regeneration in innervated and denervated mandibles (42 days after the surgery). (A1, B1) Whole-mount Alcian blue and Alizarin red staining. Arrows in A and B indicate the operated regions. Scale bars are 5 mm. (A2, B2) the section of the regenerated teeth in innervated and denervated mandibles. Scale bars are 500 μm. (A3, B3) CoMBI images of the regions shown in A1 and B1. The right mandible was processed for CoMBI imaging. Scale bars are 2 mm. (**C**) The proximal (caudal) region of A3. (**C’**) Reserved tooth buds are obvious in this higher magnification view of C. (**D**) The proximal (caudal) region of B3. (**D’**) Prevention of the formation of reserve tooth buds is apparent in this higher magnification view of D. Red arrows indicate newly forming tooth buds while green arrows indicate places where tooth bud formation was prevented. co = coronoid. de = dentary. me = Meckel’s cartilage. Scale bars C, C’, D and D’ are 3 mm. Data are representative of n = 4/group.

By day 42, tooth regeneration had somehow been restored in the denervated samples (Fig. 7, Movies 3 and 4). In the control (innervated jaw), tooth regeneration was fully completed by this time (Fig. 7A1–3,C,C’): not only functional teeth but also tooth buds for further replacement were observed (Fig. 7A3, insert). In the denervated mandibles, the dentary bone was regenerated and small teeth were observable in the regenerated region (Fig. 7B1–3). The regenerated teeth were not attached to the dentary, however, suggesting that these teeth were still non-functional (Fig. 7B1–3). Importantly, we observed differences between the denervated and intact mandibles pertaining to the tooth buds on the coronoids, where dentectomy had not been performed (Fig. 7C,C’,D,D’). As in the intact mandibles, tooth buds were observed in the basal regions of the functional teeth (Fig. 7C’), yet the number of tooth buds was apparently decreased in the denervated mandibles (Fig. 7D,D’). This suggests that nerve presence is important for tooth bud initiation not only in a bone regeneration context but also in tooth replacement.

### Fgf and Bmp expression in denervated axolotl mandible

**Figure 8:**
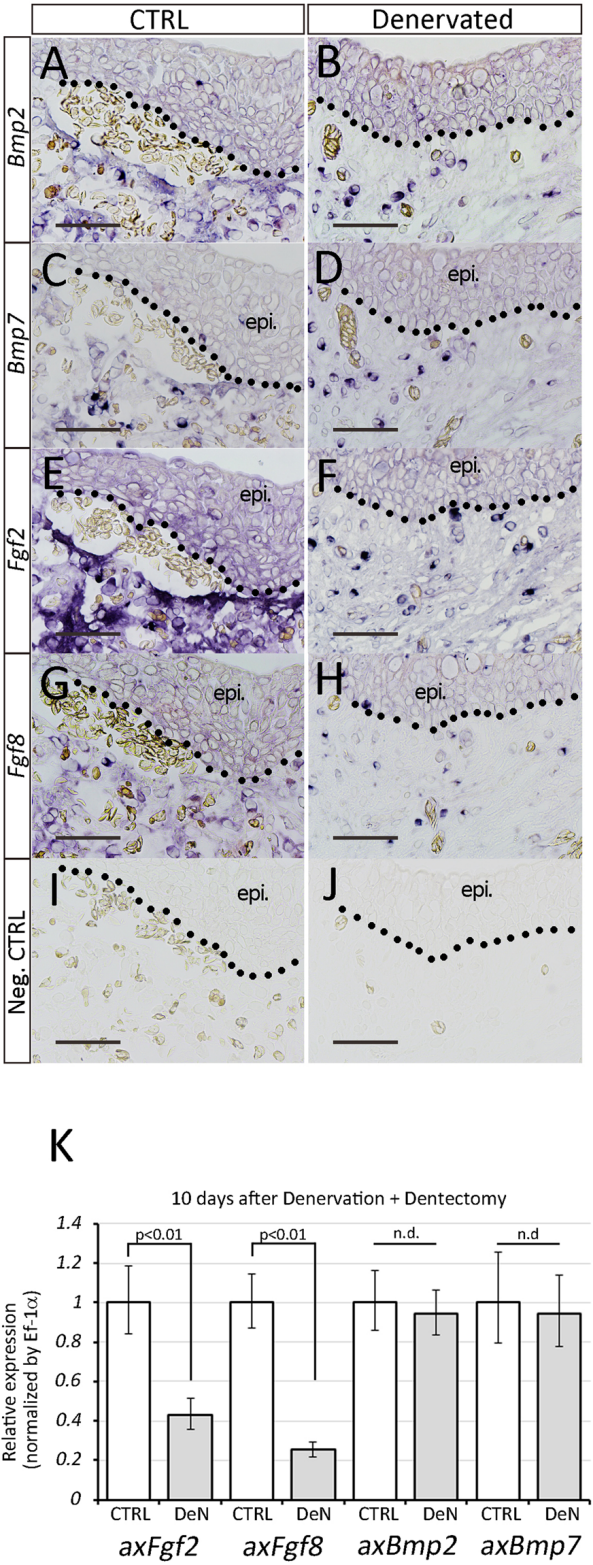
Effects of denervation on gene expression patterns in the axolotl mandible. (**A–J**) Gene expression patterns were investigated by *in situ* hybridization. (Left column) Control samples. (Right column) Denervated samples (10 days after denervation). Scale bars in A–J are 100 μm. Neg. CTRL. = negative control. epi. = oral epithelium. A–J have the same magnificatio. Data are representative of 5 axolotl mandibles. (K) Quantitative RT-PCR analysis was performed on the samples 10 days after dentectomy. Gene expression level was compared between the control and denervated samples (n = 4). Statistical analysis was performed with the *t*-test (two-tailed). DeN = denervated sample.

To investigate the effects of denervation on the expressions of these genes, we performed *in situ* hybridization. Dentectomy was performed with or without denervation, and samples were harvested at day 10. The *in situ* hybridization of *Bmp2*, *Bmp7*, *Fgf2*, and *Fgf8* genes showed relatively broad expression patterns (Fig. 8A–J). No apparent differences in *Bmp2* and *Bmp7* expression were observed between the control and the denervated mandible (Fig. 8A–D). The signal intensity of *Fgf2* and *Fgf8* appeared to be slightly weakened in the denervated mandible (Fig. 8E–H). To confirm the decrease in *Fgf2* and *Fgf8* expression, quantitative RT-PCR was subsequently performed (Fig. 8K). Denervation decreased the expression levels of *Fgf2* and *Fgf8* but had no effects on *Bmp2* or *Bmp7* expression (Fig. 8K). Therefore, both results suggest that *Fgf2* and *Fgf8* gene expression were weakened by denervation, but that there were relatively few effects on *Bmp* gene expressions.

### Tooth bud induction by exogenous Fgf and Bmp expression in denervated mandible

**Figure 9:**
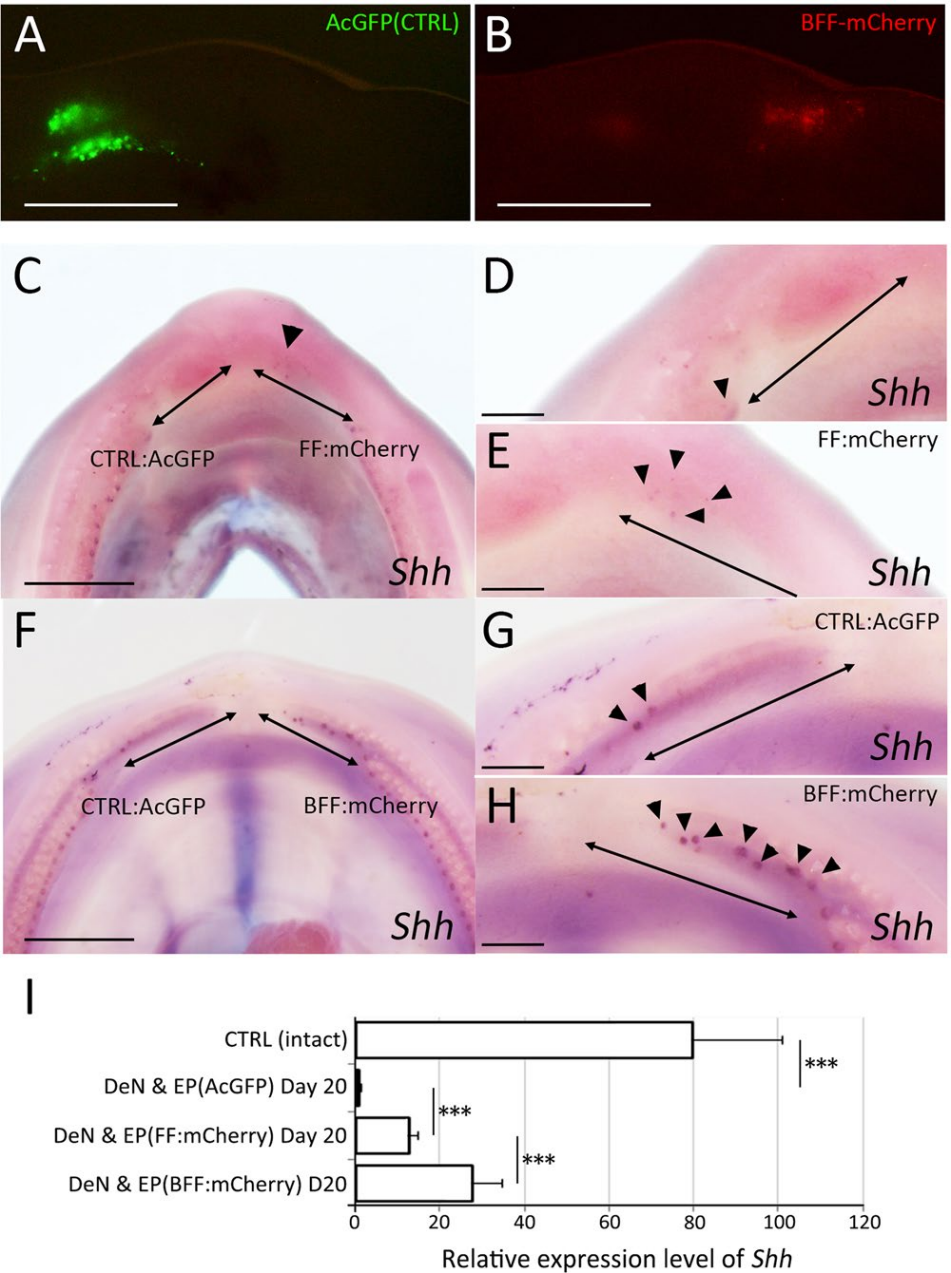
Induction of tooth regeneration by exogenous Fgf2 + Fgf8 + Bmp7. (**A,B**) Dark field views. Scale bars are 3 mm. (**A**) The control plasmids (AcGFP) were electroporated into the left side. (**B**) The Fgf8 + Fgf2 + Bmp7+mCherry plasmids (BFF-mCherry) were electroporated into the right side. (**C–H**) *Shh* expression was restored on the side where Fgf2 + Fgf8+mCherry (FF-mCherry; n = 10, **C**–**E**) or BFF-mCherry (n = 13, F–H) was electroporated. D and E are higher magnification views of the left and right sides of C, respectively. G and H are higher magnification views of the left and right sides of F, respectively. Arrows indicate the dentectomized region and arrowheads indicate *Shh* expression. Scale bars in C and F are 3 mm. Scale bars in D, E, G, and H are 1 mm. (**J**) Quantitative RT-PCR analysis was performed on the samples 20 days after dentectomy and denervation. The expression level of *Shh* was confirmed in 5 samples. Statistical analysis was performed with the *t*-test (one tailed). ***p < 0.01.

To investigate the functions of Fgf and Bmp in tooth regeneration in the axolotl mandible, we electroporated *Fgf* and/or *Bmp* genes into the denervated axolotl mandible. To achieve this, we designed the following polycistronic expression vectors: FF-mCherry, which carries *Fgf8*, *Fgf2*, and *mCherry*, and BFF-mCherry, which carries *Bmp7, Fgf8*, *Fgf2*, and *mCherry*. Electroporation was performed on day 3 and day 10 after dentectomy and denervation. The genes introduced by electroporation were considered to be temporary. However, since axolotl tooth regeneration requires a relatively long time, we performed the electroporation twice in order to provide gene expression over a sufficient period. We electroporated *Fgf* and *Bmp* on the right side of each mandible, while on the left side we electroporated AcGFP vector as a control (Fig. 9A). Clear GFP signals could be seen in the oral mesenchyme. For technical reasons it was difficult to introduce the plasmid into the overlying wound (oral) epithelium. *Shh* expression was investigated on day 20. In the control side, denervated axolotl mandible failed to induce *Shh* expressing dental placodes over a large area although a few *Shh* expressing placode(s) were seen in the proximal region (Fig. 9D,G, Table 1). FF-mCherry and BFF-mCherry gave rise to a relatively faint but confirmable mCherry signal in the oral mesenchyme (Fig. 9B). This signal gradually faded but remained detectable for a month. On the FF-mCherry or BFF-mCherry electroporated side, *Shh* spots were both clearer and much more abundant (Fig. 9C,E,F,H, Table 1). Both the proportion of successfully inducted *Shh* spots and the number of *Shh* spots were increased in BFF-mCherry transfected mandibles than in FF-mCherry transfected mandibles (Table 1). Furthermore, *Shh* expression level was confirmed by quantitative RT-PCR (Fig. 9I). The control sample, which underwent denervation and AcGFP electroporation, showed very low *Shh* expression. The electroporation with FF-mCherry or BFF-mCherry increased the *Shh* expression level. Consistent with the results from *in situ* hybridization, BFF-mCherry electroporation induced higher *Shh* expression than FF-mCherry electroporation. These results suggest that 1) exogenous Fgfs can fulfill the roles of the primary nerves and can thereby substitute for them in the induction of dental placodes in a denervated mandible and that 2) Bmp signaling plays a supportive role.

**Table 1:** Induction of Shh positive cells in the denervated mandible by regeneration inducers.

	*Shh* spot > 5	0 < *Shh* spot ≤ 5	No *Shh* spot	total
**Denervation** + **CTRL (GFP)**	0	3	8	11
**Denervation** + **Fgf2** + **Fgf8** + **mCherry**	2	3	5	10
**Denervation** + **Fgf2** + **Fgf8** + **Bmp7** + **mCherry**	6	4	3	13

## Discussion

Nerves are essential for organ-level regeneration in amphibians^23,25–27^. Their limb, tail, and gill regeneration abilities are dependent on nerve presence. Based on those findings, we focused on the role of nerves in axolotl tooth regeneration.

Hertwig published the first and fundamental study on tooth developmental events in salamanders^5^. Amphibian tooth morphology and structure have been well described previously, and the concept that amphibian tooth structures are generally similar to those of other vertebrates has been widely accepted^1^. The present study confirmed these insights. The axolotl mandible possesses haplodont dentition. As shown in Fig. 2, each axolotl tooth consists of a pulp cavity surrounded by a dentine cone. Enamel is deposited on the dentine by ameloblasts, which produce the enamel matrix^28^. As reported previously, such fundamental structures are conserved among species. Therefore, axolotl tooth development and regeneration may serve as a valuable model for tooth regeneration in mammals.

It is already widely known that axolotls are capable of tooth regeneration. A valuable description of their tooth regeneration has been published in the context of mandibular regeneration^11,12^, which has traditionally been studied through amputation of part of the upper/lower jaw. The amputated mandible grows a blastema from which the missing portion of the mandible regenerates^29^. The induced blastema then undergoes a mandibular regeneration process that includes tooth regeneration. It has remained unclear to date, however, whether jaw regeneration is an essential condition for tooth regeneration, though it was reported that tooth regeneration started before jawbone reconstitution took place^30^. Consistently, the present study demonstrated that the process whereby new teeth are induced is independent of the dentary (Fig. 6A’). Given this, tooth regeneration is likely independent of jaw regeneration. On the other hand, functional teeth were only seen attached to the dentary, implying a relationship between the axolotl tooth and the dentary. This is as expected, although no report has been published on this relationship in urodele amphibians. Though tooth regeneration may be independent of jawbone regeneration, tooth regeneration may be dependent on the oral mesenchyme. Tooth development in mammals begins with the induction of a dental placode by the oral mesenchyme^31^. This dental placode then provides positive feedback to the mesenchyme, driving tooth development further. This mesenchymal-epithelium interaction has been considered essential for mammalian tooth development. In axolotl tooth regeneration, however, this interaction remains undescribed. What controls the inductive mesenchyme and how it is induced and maintained in tooth regeneration remains an open question.

Innervation in teeth has been conserved widely among species. Our study revealed the presence of axons in teeth in the axolotl mandible. Axons were observed in the pre-invagination oral epithelium (Fig. 5E,F), the developing tooth buds (Fig. 2I,J), and the mature tooth (Fig. 2K). The axons originate from the trigeminal ganglia (Fig. 5A–C), then from many branches in the proximal region of the dentary (Fig. 5A–C). Our denervation procedure targeted major routes but was not expected to remove all of them. Insufficient removal of axons or axon regeneration may have caused the weak regeneration in the denervated mandible sample on the day 45 (Fig. 7B). The presence of nerves is necessary for successful tooth regeneration and replacement in axolotls as shown by the results depicted in Fig. 7B,D.

The present study also revealed the role of nerve presence in tooth regeneration (Fig. 6). The role of nerves in axolotl dental regeneration/development/replacement has not previously been clarified. The present results, specifically, the finding that, in the denervated mandible, invagination of the oral epithelium was delayed and *Shh* expression in the pre-invagination oral epithelium was suppressed (Figs. 6F and 9D,G), show that nerves play a role in the tooth bud induction process. Furthermore, axon presence in the pre-invagination oral epithelium follows a very suggestive pattern (Fig. 5E’,F’). Axon fibers were more densely concentrated in the regions adjacent to the invaginating dental lamina, where the next dental lamina were about to emerge. This suggests that nerves play a role in dental lamina invagination. In keeping with hypothesis, the number of newly formed tooth buds was severely decreased in the denervated mandible. Although the coronoid was not damaged in our denervation procedure, the tooth buds on the coronoid were affected by the denervation. A similar observation has been reported in a bony fish^32^, where denervation prevented tooth turnover. However, innervation and tooth bud induction are not well understood, and further investigation will be necessary on this point.

The origin of the regenerating dental lamina has been the subject of disagreement. Graver claimed that the dental lamina did not arise *de novo* from the oral epithelium but rather were regenerated from the residual lamina in the stump^14^. Clemen demonstrated that the removal of the dental lamina from the vomer of the palatine resulted in toothless bones^13^. These findings suggest that the dental lamina come from the residual oral epithelium. The present study, however, demonstrates the importance of nerve presence for tooth regeneration in the axolotl mandible. Therefore, it is possible that the results of the previous studies arose in part from unintentional nerve damage caused by the procedures. As shown in Fig. 5A–C, the nerves in this region come from the caudal direction. In Graver’s study^14^ demonstrating that proximal removal of the dentary resulted in a toothless structure, the removal of the caudal (proximal) part of the dentine would have resulted in severe loss of nerves as well. In our study, nerve loss prevented tooth regeneration in the axolotl mandible (Fig. 6B). Thus similar damage to the nerves may have been the cause of the toothless structures observed in previous studies. Moreover, we removed the oral tissues as shown in Fig. 3. The shape of the portion removed was rectangular, and the stump was located on the short side of the rectangle. We have no reason to presume that there was less migration from the long side of the rectangle and more from the short side. Considering the present results, we think that the origin of regenerating dental lamina may need to be reinvestigated.

*Fgf* and *Bmp* signaling are core regulatory cascades in limb, tail, and gill regeneration in *Ambystoma mexicanum*^20,21,24,33^. The regeneration of each of these appendages depends on the presence of nerves^34^, which express the *Fgf* and *Bmp* genes and are assumed to secrete them from the ends of axons^35^. The present study confirms that one additional tissue utilizes this common regulatory system for regeneration: the teeth, which consist of multiple tissues and cell types.

We have shown that nerves are involved in the tooth structure (Fig. 2I–K) and that the trigeminal ganglia express *Fgf* and *Bmp* genes^33^. Denervation results in the inhibition of tooth regeneration (Fig. 6). Ectopic expression of *Fgf2* and *Fgf8*, however, rescues the otherwise nerve-dependent induction of dental placodes, which is enhanced by additional *Bmp7* expression (Fig. 9E,H, Table 1). *Bmp* genes were not influenced by nerve presence (Fig. 8A,H,I), suggesting that *Bmp* expression in mandible tissues supports tooth regeneration in innervated and denervated axolotl mandibles. On the other hand, electroporation of additional *Bmp7* had clear positive effects on dental placode formation in the denervated mandible (Table 1). This implies the presence of a threshold of Bmp signaling in dental placode formation and suggests that Bmps from nerves help to overcome this threshold. The presence of a threshold of Bmp-signaling is supported by similar observations in axolotl tail regeneration^21^. In tail regeneration induced on a lateral tail wound, additional Bmp7 input to Fgf2 and Fgf8 was necessary to induce regeneration even though tail tissues express *Bmp* genes. Given these findings, it is very likely that *Fgf*s and *Bmp* are correlated with nerve presence and play important roles in tooth regeneration.

Do nerves serve as Fgf and Bmp sources? As mentioned above, the trigeminal ganglia express *Fgf* and *Bmp* genes, suggesting that the nerves from the trigeminal ganglia are able to serve as the source of Fgfs and Bmps. However, another explanation is possible with regard to tooth regeneration. Nerves may control tooth regeneration by regulating *Fgf* and *Bmp* gene expression in the axolotl mandible. Actually, denervation influences *Fgf* gene expression in the axolotl mandible (Fig. 8). As nerves are known to express many secretory molecules, unidentified factors might be responsible for regulating *Fgf* and *Bmp* genes in the axolotl mandible. Further analysis is needed to clarify this point.

It is striking that the regenerative ability of axolotl tails, limbs, gills, and teeth is controlled by a common regulatory system. Furthermore, similar regulation by *Fgf* and *Bmp* genes has been reported in the limbs of *Xenopus*, *Pleurodeles waltl*, and *Ambystoma mexicanum*^20,23^. This supports the notion of a conserved regeneration system. Recently, it has been reported that ectopic Fgf signaling can induce intercalary regeneration in chicken limb buds^36^. Likewise, mouse digit tip regeneration can be enhanced by Bmp application^37,38^. These related discoveries imply the existence of a conserved regeneration system across species. If such a conserved regeneration system exists and can be identified, it is likely to lead to innovative medical solutions.

In conclusion, axolotl can regenerate their teeth as reported previously. We renewed and deepened the insights into axolotl tooth regeneration with modern experimental technologies because no updates in this area had been made for several decades. Moreover, we clarified that tooth regeneration is regulated in a nerve dependent manner, but the same is not true of jaw bone regeneration. The nerve roles in tooth bud regeneration can be replaced by Fgf and Bmp gene expression. Such conserved nerve roles and the substitute role of Fgf and Bmp for nerves in organ regeneration in axolotls imply that the conserved mechanisms serve to regulate the regeneration of various organs.

## Materials and methods

*Animals* Axolotls (*Ambystoma mexicanum*) with nose-to-tail lengths of 8–12 cm were obtained from private breeders and housed in aerated water at 22 °C. Tubulin-GFP transgenic axolotls were kindly provided by the laboratory of Dr. E. Tanaka. In this study, the care and treatment of animals were carried out under protocols approved by the Animal Care and Use Committee of Okayama University. The animal experiments were performed following the guidelines of the animal care and use committee of the Okayama University. All efforts were made to minimize suffering according to the NIH Guide for the Care and Use of Laboratory Animals.

### Surgery

Axolotls were anesthetized with MS-222 (Sigma-Aldrich, St. Louis, MO, USA) for about 10 min (depending on the animal size) and placed on ice for 1 h in order to slow their heartbeats. Icing the animals contributes greatly to good surgical recovery. Each animal was laid out with the upper-dorsal region facing up, and dentectomy was performed in the mandibular region. A part of each animal’s mandible including the oral epithelium was dissected using forceps and scissors. About half of the dentary was dissected out, while the Meckel’s cartilage was left intact. In cases of denervation, axon dissection was performed on the same day as dentectomy. The first denervation points are indicated in Fig. 5B. The second denervation was performed on day 10; the dissection points used in this denervation are shown in Fig. 5A. After each surgery, animals were kept on ice for 2 hours to allow their wounds to heal. All animals were subsequently kept in water.

### Sectioning, histological staining, and immunofluorescence

Samples were fixed with 4% paraformaldehyde for 1 day at room temperature. In case of BrdU Decalcification with 10% EDTA was performed for 1 day. In the case of the BrdU experiment, we injected BrdU (100 μg/g bodyweight, Nakarai tesque, Kyoto, Japan) intraperitoneally 2 h before sample harvesting. Samples were embedded in O.C.T. compound (Sakura Finetek, Tokyo, Japan) following 30% sucrose/PBS treatment for approximately 12 h. Frozen sections 14 μm in thickness were prepared using a Leica (Nussloch, Germany) CM1850 cryostat. The sections were well dried under an air dryer and kept at −80 °C until use. Alcian blue and hematoxylin and eosin (HE) staining were used for histology. Immunofluorescence of the sections was performed as described in previous reports^39^. Primary antibodies – Anti-BrdU (G3G4, 1:200, DSHB, Iowa, USA), anti-acetylated tubulin antibody (sc29350, 1:500, Santa Crus Bioteth., CA, USA), anti-GFP antibody (#632377, 1:500, Takara bio. Clontech, Shiga, Japan), and anti-mCherry (#E5D8F, 1:300, R&D Systems, MN, USA) – were applied. Secondary antibodies – anti-rabbit IgG Alexa 488 (A32731, 1:500) and (A11017, 1:500) – were purchased from Invitrogen (CA, USA). Antigen retrieval for BrdU staining was performed with 10 U/ml DNase for 20 min at room temperature. Nuclei were visualized by Hoechst33342. Images were captured using an Olympus (Tokyo, Japan) BX51 fluorescence microscope.

### Block-face imaging and 3D reconstruction

Whole-mount jaw samples were fixed by 95% ethanol for 24 h. Thereafter, they were incubated in acetone for 24 h and stained for 3 h at 37 °C and then O/N at room temperature in Alcian blue and Alizarin red in 70% ethanol with 5% acetic acid (#01303 and #37154, Nakarai Tesque, Kyoto, Japan). Then, samples were well washed with tap water and re-fixed by 10% formalin (#16222, Nakarai Tesque, Kyoto, Japan). Finally, they were rinsed in tap water before clearing in 4% KOH and 20% glycerol for 24 h and then placed in graded glycerol. The stained samples were mounted in O.C.T. compound and frozen. 3D datasets of the jaw samples were obtained by the block-face imaging method. The imaging system, namely, CoMBI (correlative microscopy and block-face imaging), was constructed as described previously^16^. Briefly, a digital single-lens reflex camera (Nikon D850, Tokyo, Japan) with a macro lens (Tamron SP AF 180 mm F3.5, Saitama, Japan) and teleconverter was placed in front of the cryostat. Samples were then sectioned at a thickness of 3 μm, and block-faces were captured at every section. Serial block-face images were combined into a 3D dataset, and 3D images were reconstructed by means of volume rendering using Amira Software (version 6.4.0, Thermo Fisher Scientific, K.K., Tokyo, Japan; http://www.fei.com/software/amira-3d-for-life-sciences/) running on an iMacPro (CPU: 2.3 GHz Intel Xeon W, DRAM: 128GB 2666 MHz DDR4, and graphics: Radeon Pro Vega 64 16368 MB; Apple Japan, Tokyo).

### *In situ* hybridization

RNA probes for *in situ* hybridization were selected as previously described^39^. *In situ* hybridization was performed on each section as previously described^40^. Only *Shh* was newly cloned using the primers described in Table S1. RNA probes were subjected to alkaline hydrolysis to obtain optimal signals. Whole-mount *in situ* hybridization was performed as previously described^41^

### Electroporation

The outline of the procedure is described in Sup. Figure 1. Axolotls were anesthetized with MS222 (Sigma, MO, USA) for about 10 minutes (depending on animal size). Wipes were stuffed into each axolotl’s mouth to keep the mouth open. Plasmid solution (pCS2-EGFP, pCS2-Fgf8-Fgf2-mCherry (FF-mCherry), or pCS2-Fgf8-Fgf2-Bmp7-mCherry (BFF-mCherry), 2 μg/μl was then injected into the wounded region of the mandible. To increase the visibility of the injection, Fast green dye was added to the solution. Immediately after injection, electric pulses were applied (20 V, 50 ms pulse length, 950 ms interval, 20 times, Nepa gene, Tokyo, Japan, #NEPA21). The animals were then placed into water. Electroporation was performed at day 3 and day 10.

### RT-PCR

The denervated/innervated mandible was dissected out and mandibular bones were removed with forceps and scissors. Total RNAs for quantitative RT-PCR were prepared using TriPure reagent (Roche, Basel, Switzerland). cDNA was synthesized with PrimeScript reverse transcriptase (#2680 A, Takara, Shiga Japan). qPCR was performed using the ABI StepOne Real-Time PCR System. Data were analyzed using StepOne software version 2.1. Error bars indicate RQ_max_ and RQ_min_. The primers are described in Table S1.

## Supplementary information

Supplementary Information.

Supplementary Information2.

Supplementary Information3.

Supplementary Information4.

Supplementary Information5.

Supplementary Information6.
