# Supplementary material for: Neural regulation in tooth regeneration of Ambystoma mexicanum
Source: Sci Rep. 2020 Jun 9;10:9323. doi: 10.1038/s41598-020-66142-2 (PMC7283310; doi:10.1038/s41598-020-66142-2)
Supplement: Supplementary file 5 — Supplementary Information5. [file 41598_2020_66142_MOESM5_ESM.pdf]

Supplemental information

Neural regulation in tooth regeneration of Ambystoma mexicanum

Aki Makanae, Yuki Tajika, Koki Nishimura, Nanami Saito, Jun-ichi Tanaka, and Akira Satoh

・Supplemental Figure 1

・Supplemental Table 1

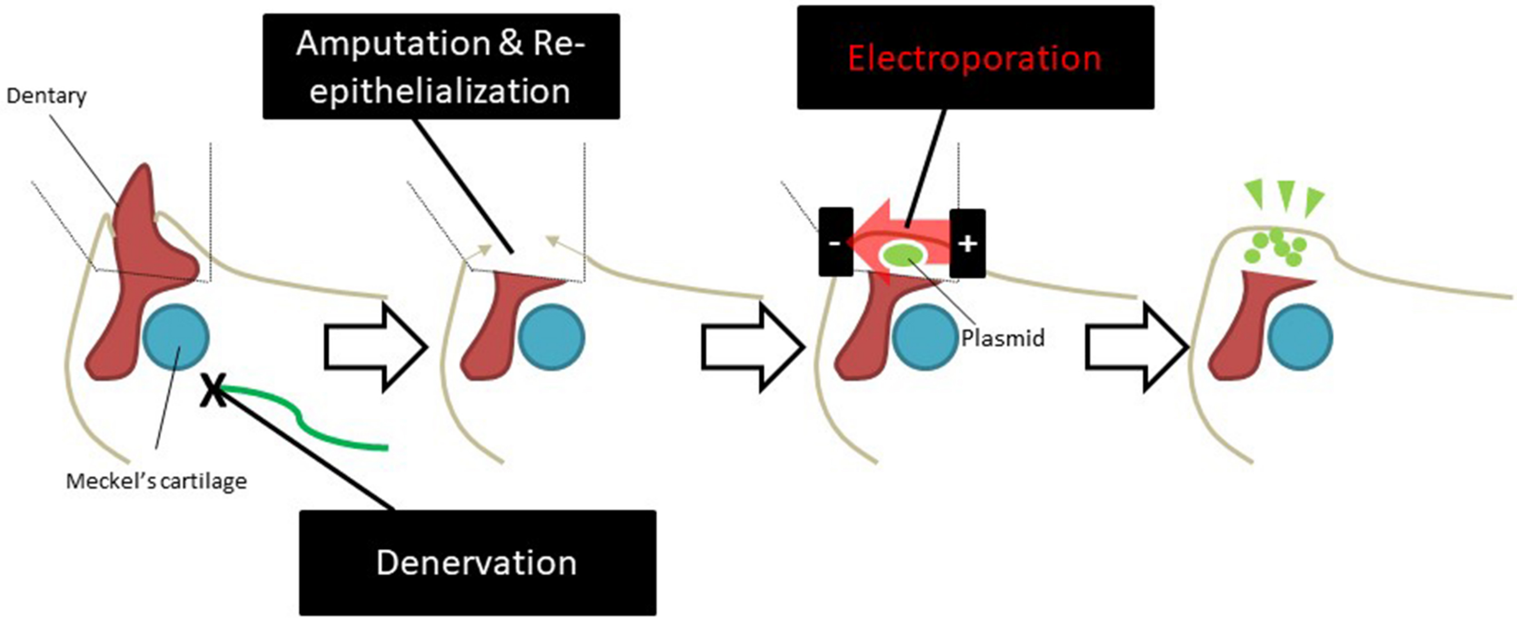

Supplemental Figure 1, Makane et al., 2020

Primer	sequence 5'→3'
Shh cloning forward primer	GAAAGGAGCGGTGCTGTTTGCGG
Shh cloning reverse primer	CTCGAGATCGATTTTTTTTTTTTTTTTTTV
Fgf2 forward primer for qRT-PCR	AGAGGAGCGACTCCTGCATA
Fgf2 reverse primer for qRT-PCR	TCCAGTTCGTTTCAGTGCCA
Fgf8 forward primer for qRT-PCR	ATCCTGGGACCAAAAGTGCT
Fgf8 reverse primer for qRT-PCR	TGCGTTCTCATGCTTCCCAT
Bmp7 forward primer for qRT-PCR	TCGGCCTTTTCGGACTTCTC
Bmp7 reverse primer for qRT-PCR	CAAACCGGAACTCTCGGTGA
Bmp2 forward primer for qRT-PCR	GAATGCCAACATCCCCAAGG
Bmp2 reverse primer for qRT-PCR	AGTGTCAGCTTTTGTGTTTGTTTT
Shh forward primer for qRT-PCR	GCTCTGTGAAAGCAGAGAACTCG
Shh reverse primer for qRT-PCR	CGCTCCGTCTCTATCACGTAGAA
EF-1a forward primer for qRT-PCR	AACATCGTGGTCATCGGCCAT
EF-1a reverse primer for qRT-PCR	GGAGGTGCCAGTGATCATGTT

Supplemetal Table S1, Makane et al., 2020
