# Supplementary material for: Neural regulation in tooth regeneration of Ambystoma mexicanum
Source: Sci Rep. 2020 Jun 9;10:9323. doi: 10.1038/s41598-020-66142-2 (PMC7283310; doi:10.1038/s41598-020-66142-2)
Supplement: Supplementary file 6 — Supplementary Information6. [file 41598_2020_66142_MOESM6_ESM.docx]

***Supplemental Figure 1***

The outline of the electroporation into the axolotl dentary. First, denervation and dentectomy were performed, followed by electroporation on day 3 and day 10 after dentectomy and denervation. Electric pulses were also applied (20 V, 50 ms pulse length, 950 ms interval, 20 times). Employing electroporation twice allowed the introduced genes to remain efficient for a longer period of time.
